# Acupuncture regulates the apoptosis of ovarian granulosa cells in polycystic ovarian syndrome-related abnormal follicular development through LncMEG3-mediated inhibition of miR-21-3p

**DOI:** 10.1186/s40659-023-00441-6

**Published:** 2023-06-12

**Authors:** Xiaohong Chen, Hengzhen He, Bingcai Long, Binli Wei, Peng Yang, Xiaoying Huang, Qian Wang, Jing Lin, Hongliang Tang

**Affiliations:** 1 Fangchenggang Hospital Affiliated to Guangxi University of Chinese Medicine, Fangchenggang, 538000 Guangxi China; 2 The First Affiliated Hospital of Guangxi University of Chinese Medicine, Nanning, 530000 Guangxi China; 3 Guangxi University of Chinese Medicine, 530200 Nanning, Guangxi China

**Keywords:** PCOS, Acupuncture, LncMEG3, miR-21-3p, Granulosa cell apoptosis

## Abstract

**Background:**

The main features of polycystic ovary syndrome (PCOS) are abnormal follicular development and ovulatory dysfunction, which are caused by excessive apoptosis of ovarian granulosa cells. Acupuncture has been shown to improve follicular development abnormalities in patients with PCOS, but its mechanism is unknown. This study hypothesized that the mechanism of acupuncture on follicular development abnormalities in PCOS patients is the inhibition of granulosa cell apoptosis through LncMEG3-mediated regulation of miR-21-3p.

**Methods:**

A PCOS-like rat model was established using subcutaneous injection of dehydroepiandrosterone (DHEA). Acupuncture was performed on rats for 15 d (CV-4, RN-3, CV-6, SP-6 and EX-CA 1). Ovarian morphology was observed by HE staining, and sex hormone and AMH levels were detected by ELISA. Primary granulosa cells were isolated from each group of rats to assess the association of acupuncture treatment, LncMEG3, miR-21-3p, and granulosa cell apoptosis in rats with PCOS.

**Results:**

LncMEG3 and miR-21-3p were highly expressed in the ovarian granulosa cells of rats with PCOS, and LncMEG3-mediated regulation of miR-21-3p was involved in the development of PCOS in rats. Silencing of MEG3 attenuated sex hormone dysregulation and ovarian histopathological changes in PCOS rats and promoted follicle cell development and maturation. In addition, silencing MEG3 increased the viability and number of granulosa cells. In addition, silencing MEG3 further inhibited early and late apoptosis of ovarian granulosa cells in PCOS rats. Acupuncture improved polycystic ovarian morphology and sex hormone levels in PCOS rats. Acupuncture intervention increased the viability and number of granulosa cells. Acupuncture intervention inhibited early and late apoptosis of ovarian granulosa cells in PCOS rats by targeting miR-21-3p via LncMEG3.

**Conclusion:**

These results suggest that acupuncture can downregulate LncMEG3, thereby targeting and regulating miR-21-3p to suppress early and late granulosa cell apoptosis and normalize their proliferation. These factors ultimately compensate for abnormal follicular development. These findings shed light on the clinical potential of acupuncture as a safe treatment for follicular developmental abnormalities in PCOS.

## Highlights

- LncMEG3-mediated inhibition of miR-21-3p regulates ovarian granulosa cell apoptosis.
- LncMEG3 and miR-21-3p are involved in the occurrence and development of PCOS-related abnormal follicular development.
- CuONPs induce co-occurrence of autophagy activation and autophagic flux blockade.
- Acupuncture can improve the sex hormone levels and follicular development in the context of PCOS.
- The underlying mechanism of acupuncture in the treatment of PCOS abnormal follicular development was revealed.

## Introduction

Polycystic ovarian syndrome (PCOS) is the most common endocrine disorder in women, with an estimated global prevalence of 6–20% of women of reproductive age [1]. It is characterized by polycystic changes in the ovaries, resulting in oligo- or anovulation [2]. Studies have found that polycystic ovary syndrome is the most likely cause of anovulation-mediated infertility and that disorders of sex hormone secretion, insulin resistance, and abnormal follicle development play an important role in the development of infertility in PCOS [3, 4]. Multiple regulatory systems are required for normal ovulation, including neuroendocrine, autocrine, and paracrine systems [5, 6]. Problems in the control of these regulatory systems may lead to dysfunctional follicular development, maturation, and ovulation, making abnormal follicular development an important pathological and pathogenic factor in PCOS [7]. It is well known that this disease is mostly treated clinically with pharmacological ovulation promotion or Western medicine surgery, but there are many adverse effects, the disease reoccurs easily after discontinuation of medication or surgery, and the long-term outcome is poor [8, 9]. Acupuncture interventions in traditional Chinese medicine (TCM) are effective and safe, and studies have found that acupuncture interventions can improve follicular development abnormalities and effectively regulate follicular development and ovarian function in patients with PCOS [10–12]. However, the understanding of the mechanisms of abnormal follicular development in PCOS treated with acupuncture still needs to be further clarified.

LncRNAs (Long non-coding RNAs) are involved in a variety of biologically important processes, such as the regulation of gene expression, dosage compensation, genomic imprinting, transcriptional activation, transcriptional interference, and nuclear transport, X chromosome silencing, and chromatin modification.LncRNAs have become an increasingly important component of the disease transcriptome and play a key role in the development of many diseases through interactions with proteins, DNA, and RNA [13]. MEG3, a lncRNA, is an imprinted gene located at locus 14q32 on the human chromosome and is significantly expressed in embryonic development and in a variety of normal cells [14]. This suggests that LncMEG3 may be closely related to the development of disease and is a potential target for future disease diagnosis and treatment. It is well known that high miR-21 expression is closely associated with the induction of ovarian autocrine and paracrine dysfunction and apoptosis. Abnormal miR-21 expression in the serum of PCOS patients is involved in the process of PCOS [15, 16], and miR-21 plays a role in the apoptotic response [17]. It has been demonstrated that acupuncture inhibits the expression of Bcl-2, an apoptosis-related indicator, and miR-21 in granulosa cells of ovarian cancer rats [18], suggesting that acupuncture may have a regulatory effect on apoptosis and the miR-21 gene. However, it is still unknown whether the modulatory and inhibitory effects of acupuncture on the miR-21 gene and granulosa cell apoptosis in PCOS are dependent on the granulosa cell MEG3 gene.

Our previous studies have demonstrated that acupuncture can regulate the abnormal expression of LncMEG3 in granulosa cells and inhibit granulosa cell autophagy [19]. It is suggested that the granulosa cell MEG3 gene plays an important role in the treatment of PCOS with acupuncture. However, the role of the granulosa cell MEG3 gene in abnormal follicle development in the context PCOS and acupuncture treatment remains unclear. Granulosa cells are the main functional cells of the ovary, and their proliferation and differentiation directly affect the functional activities of the ovary, such as follicle growth initiation, development, ovulation, luteal formation, and steroid hormone secretion. The role of granulosa cell apoptosis in PCOS has been an important area of research [20]. Granulosa cell apoptosis occurs in all phenotypes of PCOS, and inhibition of granulosa cell apoptosis can alleviate the clinical manifestations of PCOS with insulin resistance, hyperandrogenemia, disturbance of sex hormone secretion, and abnormal follicular development [21]. In mammalian cells, mitochondria play an important role in the induction of apoptosis, which is regulated by members of the Bcl-2 family [22]. The mitochondria-mediated pathway is as follows: Stress and other apoptotic stimuli trigger the migration of Bcl-2 family members such as Bax into the mitochondria. This event leads to the release of cytochrome C from the mitochondria into the cytoplasm [23], a process sometimes accompanied by loss of mitochondrial membrane potential and loss of stability of the outer layer of the mitochondrial membrane (early in apoptosis, involving mainly changes in some intracellular proteins). In the presence of dATP, cytochrome C binds to Apaf-1 to form the Apaf-1/cytochrome C/dATP complex, the apoptosome [24], which activates pre-caspase-9 and then causes the activation of effector caspases such as caspase-3. Caspase-3 has an important role in the proteolytic cleavage of ICAD (DFF45) into CAD (DFF45). Caspase-3 has an important role in the cleavage of ICAD (DFF45) into CAD (DFF40), which promotes the breaking of DNA into separate nucleosomal units (mid- to late-stage apoptosis is accompanied by DNA breakage and the formation of apoptotic vesicles) [25]. Therefore, our study assessed the expression of apoptotic proteins (including cleaved caspase-3, Bax, and Bcl-2), mitochondrial membrane potential (JC-1), and TUNEL staining to detect early, mid, and late-stage apoptosis.

Anti-Müllerian hormone (AMH) mainly inhibits the entry of the primordial follicle into the growth phase and is most highly expressed in small sinus follicles [26]. In PCOS, the main manifestation is abnormal follicular development leading to persistent anovulation, and high LH and LH/FSH ratios and insulin levels in PCOS patients promote androgen secretion. High levels of AMH inhibit the conversion of testosterone (T), the main component of androgens, to estrogen by reducing the sensitivity of granulosa cells to FSH and inhibiting the expression of P450 aromatase; in addition, E2 levels are reduced, resulting in a build-up of androgens in the body, which in turn promotes the production of AMH, creating a vicious cycle that ultimately leads to a blocked selection of dominant follicles and stagnation of follicle development in the small sinus follicle stage [27]. Numerous studies have confirmed that serum AMH concentrations are two to three times higher in patients with PCOS than in normal women. This is because the primary cause of anovulation in PCOS is an increased number of antral follicles, diminished follicular selection, and the absence of dominant follicle production, suggesting dysfunctional growth and development of early follicles in the ovary [28–30]. Therefore, in this project, AMH, FSH, LH, E,2 and T were chosen as indicators of efficacy in evaluating PCOS.

Therefore, we hypothesized that the restoration of granulosa cell MEG3 expression and potential MEG3 targeting of miR-21 involved in granulosa cell apoptosis are important mechanisms of acupuncture in the treatment of PCOS; neuronal MEG3 in granulosa cells may be an important target involved in acupuncture treatment of PCOS. In this study, we investigated the mechanism of action of acupuncture in treating abnormal follicle development in PCOS based on the understanding of PCOS based on TCM theory and the analysis of acupuncture points based on clinical experience combined with meridian selection [31] and verified the effects of acupuncture on reproductive endocrine levels, ovarian granulosa cell apoptosis,miR-21-3p and LncMEG3 expression in PCOS rats. To test our hypothesis, we first assessed the expression of MEG3 and miR-21-3p in granulosa cells of dehydroepiandrosterone (DHEA)-treated mice and determine whether the expression levels of MEG3 and miR-21-3p were positively correlated. To evaluate the role of MEG3-mediated regulation of miR-21-3p in granulosa cells and acupuncture in the treatment of PCOS, a dual-luciferase reporter assay was used to verify the targeting relationship between MEG3 and miR-21-3p. Finally, to investigate the possible relationships between the MEG3 and miR-21-3p genes, granulosa cell apoptosis, acupuncture treatment, and abnormal follicular development in PCOS, an experiment was designed to downregulate MEG3 in granulosa cells using a lentivirus carrying MEG3 shRNA. To our knowledge, this is the first study of the targeting relationship between ovarian granulosa cell MEG3 and miR-21-3p in a rat model of PCOS, highlighting the importance of MEG3, miR-21-3p and granulosa cell apoptosis, especially ovarian granulosa cell MEG3, in the treatment of abnormal follicular development in PCOS by acupuncture.

## Materials and methods

### Experimental animals

Experimental animals were selected from 3-week-old female Sprague‒Dawley (SD) rats of breeding age. Animals were obtained from Hunan Slaughter Experimental Animal Co., Ltd. (Hunan, China) [licence number: SYXK (Gui) 2019-0004]. They were housed under a 12-h light/dark cycle with a room temperature of 20 ± 0.5 °C and humidity between 30.0% and 50.0%, with free access to food and water. Prior to experimental manipulation, rat nests and cages were changed daily, and the rats were habituated indoors for at least one week after entering the laboratory. The experimental design was a single study approved by the local ethics committee of Guangxi University of Chinese Medicine, People's Republic of China (animal ethics approval number: DW20220621-129).

### DHEA-induced PCOS model

The PCOS rat model was induced according to the method of Lee et al. [32–34], and to establish the PCOS rat model, 6 mg/100 g of DHEA was injected into each rat, for 15 consecutive days, where DHEA was dissolved in 0.3 mL castor oil (MedChemExpress, China). Blank controls were injected subcutaneously on the back for 15 d continuously with 0.3 mL castor oil during the same period. Sixty PCOS rats were successfully verify by vaginal cytology, plasma hormone profile and ovarian histology on day 10 of the oestrous cycle [22].

### Grouping and lentivirus injection

Sixty rats with PCOS were randomly divided into six groups (n = 10/group): dehydroepiandrosterone (DHEA, MedChemExpress, China) PCOS group, small hairpin RNA (shRNA) negative control group (sh-NC), MEG3-shRNA lentivirus (sh-MEG3) group, acupuncture group (PCOS + A), sham acupuncture group (PCOS + SA) and western medicine group (PCOS + W). Rats in the sh-NC and sh-MEG3 groups were fasted for 12 h. Lentiviral injections were performed after the PCOS rat model [17]. After anaesthesia, the rats were fixed in lateral position by electric stereotaxic positioning, and 1–2 cm incisions were made in the bilateral abdomen. The skin-fascia-muscle were sequentially cut by scissors or bluntly separated, white moist fatty masses could be seen, and pink bright cauliflower-like ovaries were visible by ridding the folds of fatty masses. shRNA-NC or MEG3-shRNA lentivirus (Shanghai Gikai Genetic Pharmaceutical Technology Co., Ltd., Shanghai, China, viral titer of 5 × 10^8^ transduction units/ml) was injected into rat ovaries with a 10 μl syringe. The needle was injected slowly and kept in place for 5 min. Each ovary was injected twice at different sites with 10 μl each. In total, 40 μl of lentivirus was injected into each rat. An appropriate amount of intramuscular penicillin (Sichuan, China, 80,000 units/ml) was applied to prevent infection, and suturing was sufficient.

### Acupuncture treatment

As previously described [51], during the acupuncture treatment, mice were placed in a specially designed rat immobilizer with the rat in the supine position and the fixed trunk kept immobile, while the head and limbs were free to move. "Guan Yuan" (located approximately 25 mm below the umbilicus) and "Zi Gong" (located approximately 30 mm below the umbilicus) were selected according to the "Animal Acupuncture Atlas" developed by the Experimental Acupuncture Branch of the Chinese Acupuncture and Moxibustion Society. San Yin Jiao" (located approximately 30 mm below the umbilicus and 20 mm below the umbilicus) and "San Yin Jiao" (located 10 mm straight up from the tip of the inner ankle of the hind limb); Zhong Ji, Guan Yuan and Qi Hai are the Ren Chakra of the body, which are 4", 3" and 1.5", respectively. They are located below the midline of the umbilical cord. The localization of Guan-Yuan in the rat has been clarified. Using this as a conversion criterion, the human acupoints were transplanted to the rat. The distribution of Qi Hai, Guan Yuan, and Zhong Ji in the rat was determined to be 12.5 mm, 25 mm, and 34 mm below the umbilicus, respectively. Acupuncture was performed at the above acupuncture point locations, and disposable silver needles (Hwato, China, 0.25*25 mm) were inserted 5–8 mm into each point and positioned by slight muscle tremors. The needles were left in place for 30 min each day for 15 days. After successful modelling of the rats in the sham acupuncture group, the same restraint method was used, and acupuncture was performed on the skin of the non-acupuncture points (Guan Yuan, Zhong Ji, Qi Hai, Sanyin Jiao, and 5 mm outside the uterus point), and silver needles were inserted 5–8 mm and positioned by slight muscle tremor. The needles were left in place for 30 min each day for 15 days.

### Morphological observation of rats

(1) Body weight monitoring of rats. Starting from day 1 of the intervention, the rats were weighed on electronic scales at 9:00 a.m. daily to detect the body weight of the rats, and the dynamic changes in the body weight of the rats were observed and recorded. (2) Observation of rat ovarian morphology. Rats were injected intraperitoneally (3% pelltobarbitalum natricum. Merck, Germany, 0.03 ml/100 g) to induce anaesthesia, and after successful anaesthesia and extraction of blood samples from rats, ovarian tissues were collected from the left or right ovary of each rat. The tissue was fixed in 4% paraformaldehyde solution, the embedded tissue was embedded in paraffin, and the final section thickness was 4 μm. The sections were then dehydrated in a graded ethanol series of 95%, 90% and 70%, cleared in xylene and stained with haematoxylin and eosin. Finally, sections were observed using a Leica DMi1 microscope (Leica, Germany).

### Collection of primary granulocytes

Granulosa cells from rat ovaries were extracted according to a previous study [19], and anaesthesia was induced by intraperitoneal injection (3% pelltobarbitalum natricum. Merck, Germany, 0.03 ml/100 g). A sterile area was established, and the abdomen of the rats was entered, with the bilateral ovaries of the rats located beneath their bilateral kidneys. They were isolated, and ovarian tissue was obtained by repeatedly washing and removing ovarian vessels, surface membranes and other tissues with prewarmed phosphate-buffered saline (PBS) containing double antibodies. Each group of rats was randomly assigned 10 rat unilateral ovaries to be extracted with primary granulosa cells, while the stripped rat ovaries were punctured with a 1 mL syringe needle to puncture the follicles, and the granulosa cells were transferred into DMEM/F12 culture by repeated blowing, and the ovaries were cut with ophthalmic scissors, added with 0.25% collagenase 1 m at 37 °C, 5% CO2 incubator for 1 h, and added to DMEM/F12 (15% foetal bovine haematocrit). F12 (15% foetal bovine serum) culture medium to terminate the digestion. The cells were filtered through a 220 mesh cell sieve, placed in a 50 ml centrifuge tube and centrifuged at 1000 rm/5 min, and the supernatant was discarded to collect the cells. The loose cell mass deposited at the bottom of the centrifuge tube was added to DMEM/F12 (15% foetal bovine serum, Gibco, New York, NY, USA + double antibody, Solarbio, Beijing, China) and incubated at 37 °C for 24 h in a 5% CO2 incubator and then changed once to remove unadhered cells and every other day thereafter.

### Determination of sex hormones (T, E2, LH, FSH) and AMH by enzyme-linked immunosorbent assay (ELISA)

Sex hormones (T, E2, LH, FSH) and AMH in rat serum were determined using an ELISA kit (Elabscience, Wuhan, China) with steps according to the manufacturer's instructions by placing rat serum frozen at − 80 °C for 2 h at room temperature or overnight in a 4 °C refrigerator. Briefly, rat serum samples were assayed on a 96-well enzyme-labelled plate [26]. First, 100 µL of standards at different concentrations were added to the standard wells, 100 µL of the sample to be tested was added to the sample wells, and 100 µL of the enzyme standard reagent was added to each well except the blank wells, and the plate was sealed and incubated at 37 °C for 90 min. After dilution of the washing solution, add washing solution to each well, let stand for 1 min and discard, pat dry, repeat 3 times; add 100 µL each of substrate A and B to each well, incubate for 15 min at 37 ℃ and avoid light, terminate; measure absorbance at 450 nm in each well and calculate sample concentration.

### Protein extraction and Western blot analysis to measure the expression of caspase-3, cleaved caspase-3, Bax and Bcl-2 in ovarian granulosa cells

Cultured primary ovarian granulosa cells were manipulated on ice, and cells were homogenized at a ratio of 100:1. RIPA lysis buffer (Beyotime, Shanghai, China), PMSF (Sangon Biotech, Shanghai, China) and protease inhibitor (MCE, China) were added to extract ovarian granulosa cell protein concentrations, and BCA assay kits (Beyotime, Shanghai, China. China) to determine ovarian granulosa cell protein concentrations, and the cooked protein samples were subjected to SDS‒PAGE, membrane transfer, 10% skim milk closure, and the addition of goat anti-rabbit caspase-3 polyclonal antibody (1: lysed caspase-3 polyclonal antibody (1:700, Cell Signaling Technology, Danvers Massachusetts, USA), Bax polyclonal antibody (1:2000, Proteintech, Wuhan, China), Bcl-2 polyclonal antibody (1:2000, Proteintech, Wuhan, China) and β-actin (1:5000, Proteintech, Wuhan, China). Proteintech, Wuhan, China) were refrigerated overnight at 4 ℃. The membranes were washed three times with TBST, incubated with horseradish peroxidase-labelled secondary antibody (goat anti-rabbit IgG, 1:5000, Proteintech, Wuhan, China), and washed with TBST, and the target protein bands were detected with an enhanced chemiluminescence detection system (Millipore, Burlington, MA, USA). The greyscale values were analysed using ImageJ software, and the results were recorded.

### Dual-luciferase reporter gene assay

The MEG3 fragment containing the specific miR-21-3p binding site was cloned into the pmirGLO dual-luciferase expression vector (Promega, Madison, WI, USA) to produce pmirGLO-MEG3-Wt. In addition, the same binding site of miR-21-3p in MEG3 was mutated to construct pmirGLO-MEG3-Mut. pmirGLO-MEG3-Wt was transfected with miR-21-3p mimics into miR-NC-, pmirGLO-MEG3-Mut-or pmirGLO vector-transfected cells. Approximately 48 h after transfection, luciferase activity was measured using a dual-luciferase reporter assay system (Promega).

### Real-time PCR

RNA was extracted from rat ovarian granulosa cells using the Eastep® Super Total RNA Extraction Kit (Promega, Shanghai, China). RNA (2–6 µl) was reverse transcribed into cDNA using the Superscript Reverse Transcriptase Kit (Takara Bio, Beijing, China) as a template. was reverse transcribed to cDNA. cDNA samples were subjected to real-time PCR using the TB Green® Premix Ex Taq™ II PCR kit (Takara Bio, Beijing, China) on a Light Cycler/Light Cycler480 system (Roche, Basel, Switzerland). The PCR conditions were as follows: 95 °C predenaturation for 30 s, 95 °C for 30 s, 60 °C for 10 s, 1 plate reading at the end of extension × 40 cycles, annealing at 50 °C for 1 min, and melting curve analysis at the end of the cycle to assess the specificity of the PCR products, whose relative expression levels were calculated as (2^−△△ct^). The primer sequences are shown in Table 1.

**Table 1:** PCR primer sequences

Gene name	Primer sequences (5’ to 3’)
LncMEG3	5′-GCTGGGTCGGCTGAAGAAC-3′ 5′-TGGCTGTGGAGGGATTTCG 3,
miR-21-3p	5′-AGCAGTCGATGGGCTGTCA-3′
Bcl-2	5′- CATGCGACCTCTGTTTGATTT-3′ 5′-TCACTTGTGGCCCAGGTATG-3′
bax	5′-GATGAACTGGACAACAACATGGA-3′ 5′-CAAAGTAGAAAAGGGCAACCAC-3′
caspase-3	5′-GCAGCAGCCTCAAATTGTTGAC-3′ 5′-TGCTCCGGCTCAAACCATC-3′
β-actin	5′-GGAGATTACTGCCCTGGCTCCTA-3′ 5′-GACTCATCGTACTCCTGCTTGCTG-3′

F forward, R reverse, LncMEG3 long noncoding RNA maternally expressed gene3, miR-21-3p microRNA-21-3p, Bcl-2 BCL2 mRNA, Bax BAX mRNA, caspase-3 Casp3 mRNA

### TUNEL assay

A TUNEL kit (Elabscience, Wuhan, China) was used to detect apoptosis: ovarian tissues (fixed in 4% paraformaldehyde for 24 h) were dewaxed, hydrated, and treated with proteinase K, and the tissues were added to TdT equilibration buffer working solution. Tissue was added to TdT equilibration buffer working solution, reacted with labelling working solution, nuclei were restained with DAPI working solution, sealed with a sealant containing an anti-fluorescence quencher and observed under a fluorescence microscope in DNase I-treated samples with DNA strand breaks (late apoptotic cells) showing intense fluorescent staining (green). Nuclei were counterstained with DAPI (blue). Three random fields of view were photographed under the microscope, and the apoptotic index (green fluorescence value) was calculated using ImageJ software.

### Mitochondrial membrane potential (JC-1)

Groups of 25 T primary pellet cells were cultured in 6-well plates, and one well of the 6-well plate was cultured for 3–4 days. The culture fluid was aspirated, and 1 ml of cell culture medium was added after washing the cells once with PBS. The cell culture medium can contain serum and phenol red. One millilitre of JC-1(Beyotime, Shanghai, China) staining working solution was added and mixed thoroughly. Incubate the cells for 20 min at 37 °C in a cell incubator. After incubation at 37 °C, the supernatant was aspirated and washed 2 times with JC-1 staining buffer. Add 2 ml of cell culture medium.Finally, the cells were observed under a fluorescence microscope, and the apoptotic index (green fluorescence value) was calculated using ImageJ software.

### Statistical analysis

Statistical analyses were performed using Prism8 software packages and SPSS statistical package (version 20.0, IBM Armonk, NY, USA). All data are reported as the mean ± standard deviation ($\overline{X}$±s). One-way analysis of variance (ANOVA) was used for the comparison of measurement data between multiple groups, and two-by-two comparisons between groups were performed with a t test for those with equal variance and a rank-sum test for those with unequal variance. Differences were considered statistically significant at *P* < 0.05.

## Results

### High expression of LncMEG3 and miR-21-3p in ovarian granulosa cells found in rats with PCOS

**Fig. 1:**
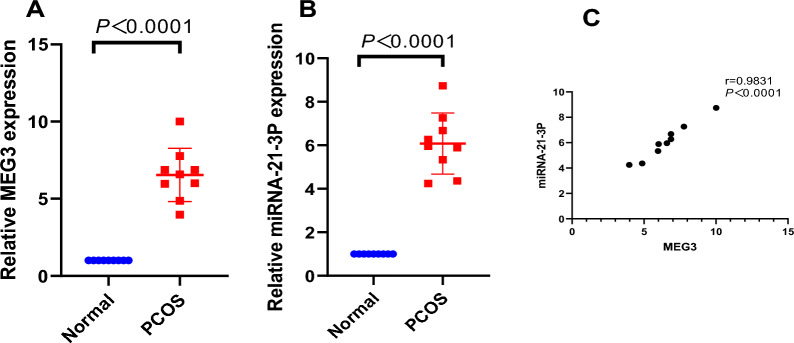
MEG3 and miR-21-3p expression are high in rats with PCOS. **A** MEG3 expression in ovarian granulosa cells of rats with PCOS by RT‒qPCR; **B** miR-21-3p expression in ovarian granulosa cells of rats with PCOS by RT‒qPCR; **C** Pearson analysis of the relationship between MEG3 and miR-21-3p in ovarian granulosa cells of rats with PCOS

LncMEG3 is involved in the progression of human diseases by regulating miR-21-3p, but its role in PCOS is unclear. In this study, the expression of LncMEG3 (Fig. 1A) and miR-21-3p (Fig. 1B) in ovarian granulosa cells of PCOS rats was detected by RT‒qPCR and Western blot analysis, and the results showed that the expression of LncMEG3 and miR-21-3p was increased in the PCOS group compared with the Normal group (both *P* < 0.0.001). To investigate the correlation of the LncMEG3/miR-21-3p axis in PCOS, we applied the Pearson test to analyse the relationship between LncMEG3 and miR-21-3p expression, and the results showed (Fig. 1C) a positive correlation between LncMEG3 expression and miR-21-3p expression in ovarian granulosa cells of PCOS patients (r = 0.9831, *P* < 0.0001).

### LncMEG3 targets and regulates miR-21-3p

**Fig. 2:**
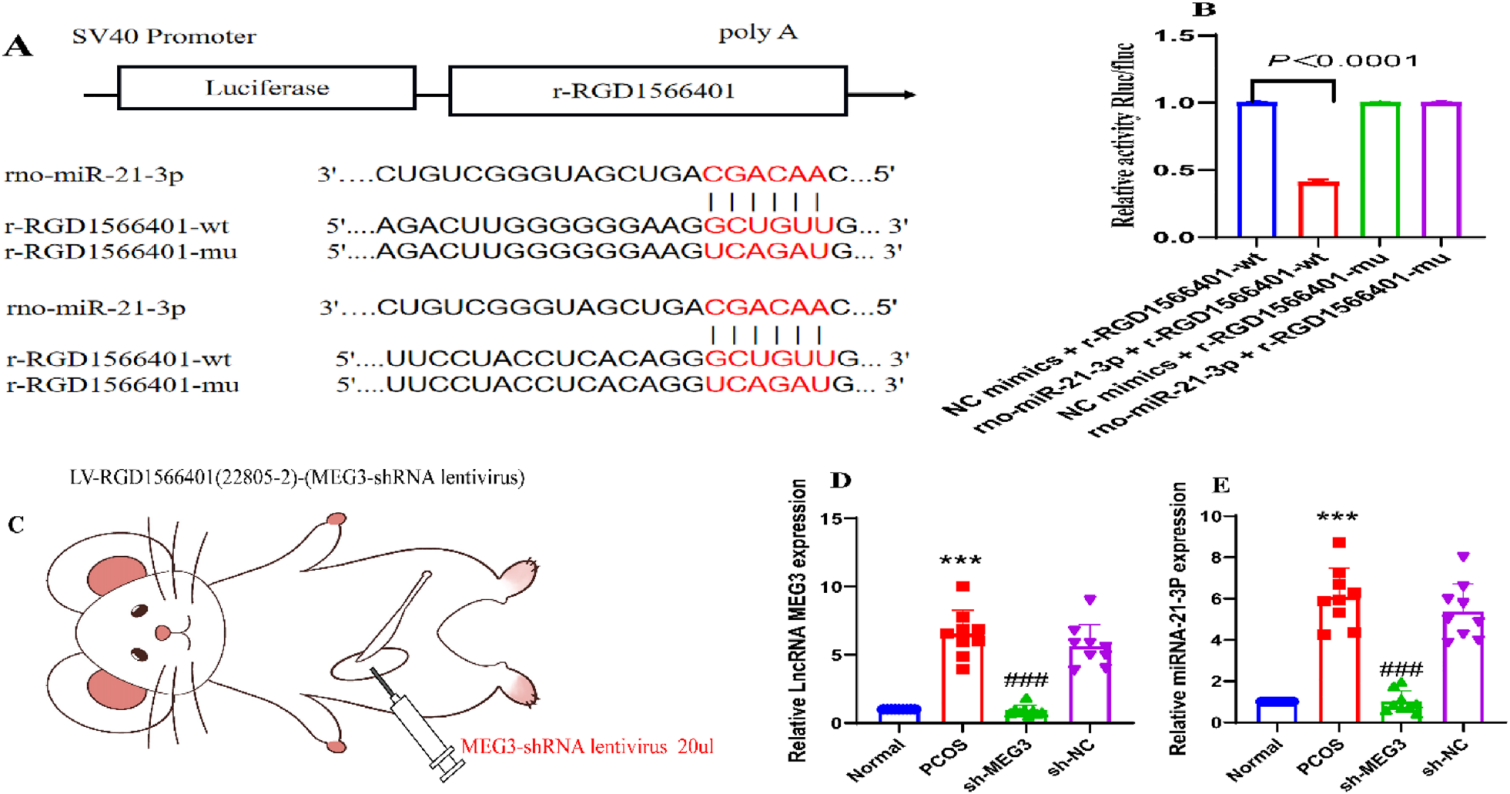
MEG3 binds to miR-21-3p, a direct target gene of miR-21-3p in PCOS rats. **A** Putative complementary binding site of miR-21-3p and MEG3; **B** Fluorophore activity assay verified the targeting relationship between MEG3 and miR-21-3p; **C** Experimental strategy for the inhibition of ovarian MEG3 expression by viral injection; **D** Detection of LncMEG3 by RT‒qPCR in ovarian granulosa cells of PCOS rats; **E** The expression of miR-21-3p was detected by RT‒qPCR in the ovarian granulosa cells of PCOS rats. A-E, N = 6. Data reflect all measurements and are expressed as the mean ± standard deviation. A t-test was used for statistical analysis between two groups, and one-way ANOVA and Tukey's post hoc test were used for statistical analysis between multiple groups. ***P < 0.0001 compared to the normal group; ^###^ *P* < 0.0001 compared to the sh-NC group

To further analyse the relationship between LncMEG3(RGD1566401) and miR-21-3p and the relationship between LncMEG3 and miR-21-3p in PCOS, the binding sites between LncMEG3 and miR-21-3p were analysed, and pmirGLO-RGD1566401-WT and pmirGLO-RGD1566401-Mut plasmids were constructed (Fig. 2A). Dual-luciferase reporter gene analysis showed that rno-miR-21-3p significantly decreased the luciferase activity of the r-RGD1566401-WT construct compared with that of the NC construct (*P* < 0.0001), indicating a binding interaction between the two sites; after mutation, rno-miR-21-3p failed to decrease r RGD1566401-MUT luciferase activity (*P* > 0.05), indicating that the mutation was successful. This result suggests that miR-21-3p is a target of MEG3 (Fig. 2B).

**Fig. 3:**
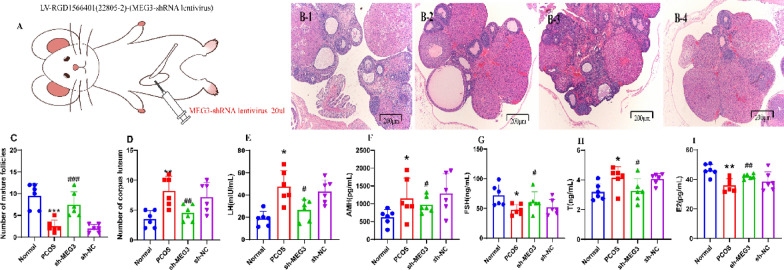
Silencing MEG3 ameliorates abnormal sex hormone levels and ovarian histopathological changes in PCOS rats. **A** Experimental strategy for the inhibition of ovarian MEG3 expression by viral injection;** B** HE staining was used to observe pathological changes in the ovaries of PCOS rats after MEG3 downregulation (scale bar = 200 μm); **C** Number of mature follicles in the ovarian tissue of PCOS rats after MEG3 downregulation; **D** Number of corpus luteum structures in the ovarian tissue of PCOS rats after MEG3 downregulation; **E** Serum secretion of LH in PCOS rats after silencing MEG3; **F** Serum secretion of AMH in PCOS rats after silencing MEG3; **G** Serum secretion of FSH in PCOS rats after silencing MEG3;**H** T secretion in the serum of PCOS rats after MEG3 silencing; **I** E2 secretion in the serum of PCOS rats after MEG3 silencing. A-I, N = 6. These data are expressed as the mean ± standard deviation, *P < 0.05, **P < 0.01, ***P < 0.0001 compared with the normal group; ^#^*P* < 0.05, ^##^*P* < 0.01, ^###^*P* < 0.0001 compared with the sh-NC group, and statistically analysis using one-way ANOVA and Tukey's post hoc test. e2 stands for oestradiol, AMH stands for anti-Mullerian hormone, T stands for testosterone, FSH stands for follicle-stimulating hormone, and LH stands for luteinizing hormone. 1: Normal group, 2: PCOS group, 3:sh-MEG3 group, 4:sh-NC group

**Fig. 4:**
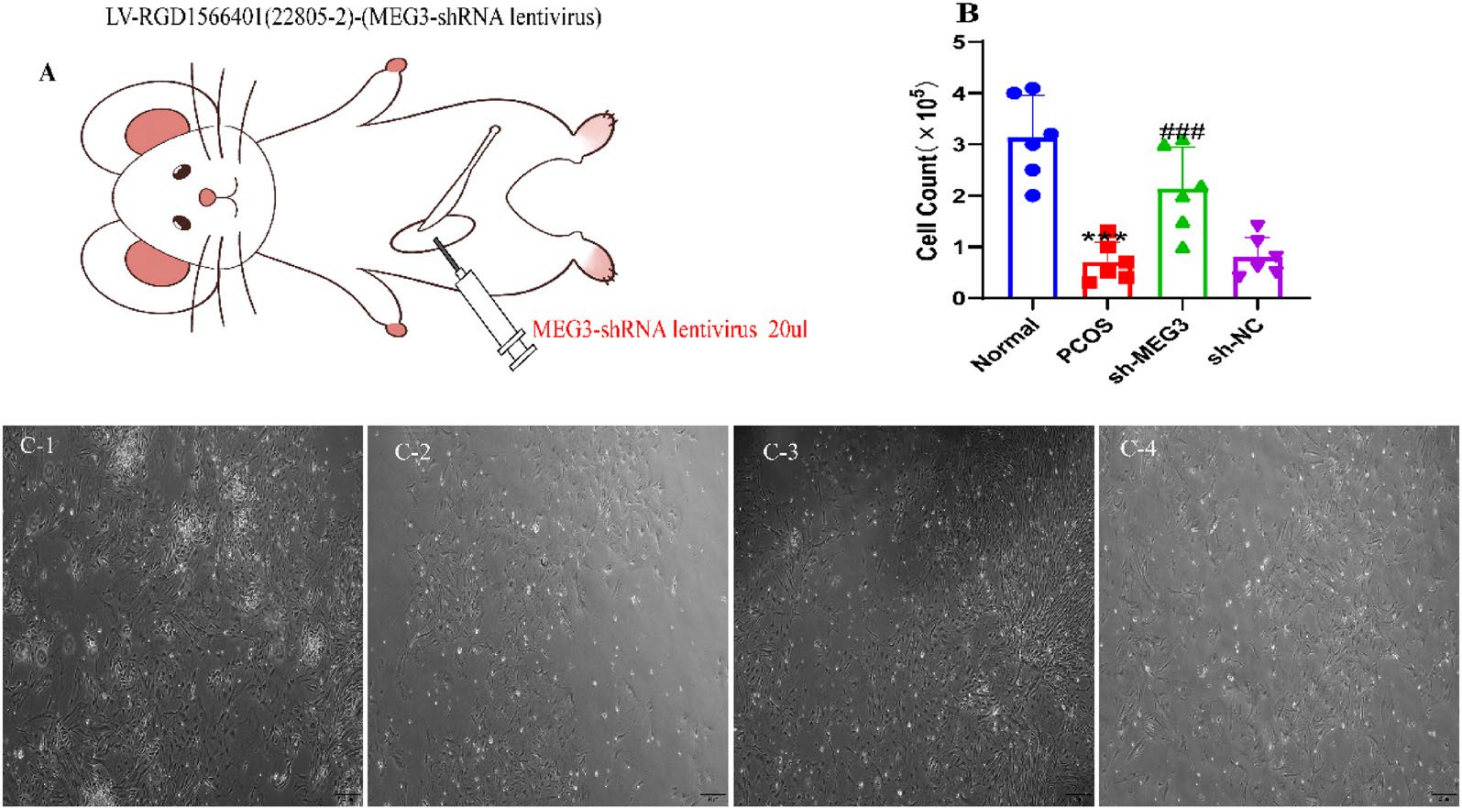
Silencing MEG3 increases the number of granulosa cells. **A** Experimental strategy for the inhibition of ovarian MEG3 expression by viral injection; **B** MEG3 downregulation of ovarian granulosa cells in PCOS rats; **C** Primary granulosa cells were extracted and cultured to observe cell number; The number of granulosa cells in PCOS rat ovaries were significantly increased after MEG3 downregulation (scale bar = 100 μm). A-C, N = 6. These data are expressed as the mean ± standard deviation, ***P < 0.0001 compared to the normal group; ^###^*P* < 0.0001 compared to the sh-NC group and were statistically analysis using one-way ANOVA and Tukey's post hoc test. 1: Normal group, 2: PCOS group, 3:sh-MEG3 group, 4:sh-NC group

**Fig. 5:**
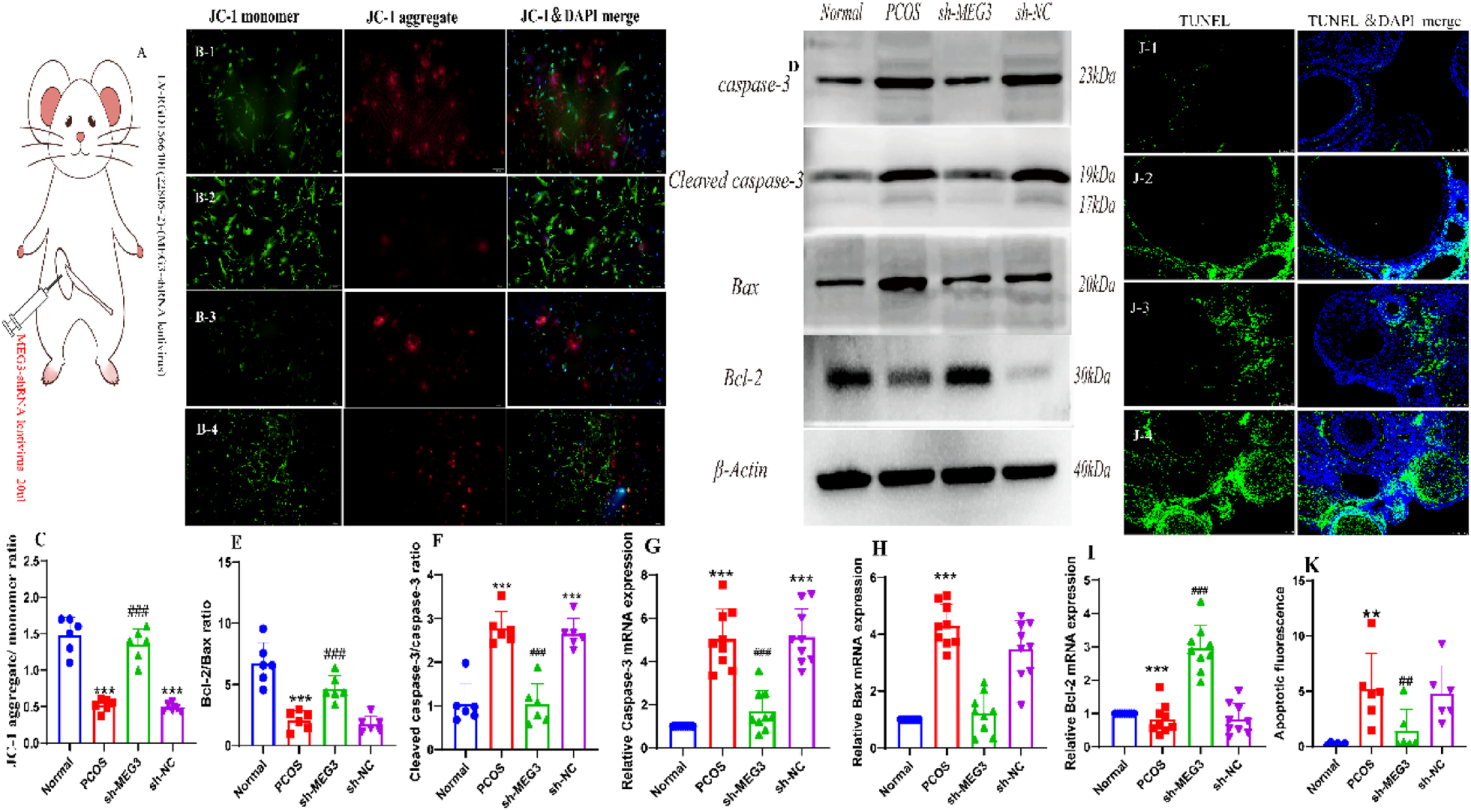
Silencing of MEG3 inhibited apoptosis in PCOS rat ovarian granulosa cells. **A** Experimental strategy for the inhibition of ovarian MEG3 expression by viral injection; **B** The JC-1 method was used to detect the early stage of apoptosis in PCOS rat ovarian granulosa cells after MEG3 downregulation (scale bar = 100 μm):at high mitochondrial membrane potential, which can produce red fluorescence, at low mitochondrial membrane potential, which produces green fluorescence, and the blue fluorescent DAPI dye labels the nucleus, and Merge is a superimposed image showing the three fluorescent signals; **C** The ratio of JC-1 aggregate (red) to monomer (green) intensity in myotubes, Cell experiments were independently repeated three times;** D** cleaved caspase-3, caspase-3, Bax and Bcl-2 protein bands in PCOS rat ovarian granulosa cells after MEG3 downregulation; **E** Western blotting to detect the Bcl-2/Bax ratio in PCOS rat granulosa cells after MEG3 downregulation in granulosa cells; **F** Western blotting to detect cleaved caspase-3/caspase-3 protein expression in PCOS rat granulosa cells after MEG3 down regulation; **G** Detection of caspase-3 by RT‒qPCR in ovarian granulosa cells of PCOS rats; **H** Detection of Bax by RT‒qPCR in ovarian granulosa cells of PCOS rats; **I** Detection of Bcl-2 by RT‒qPCR in ovarian granulosa cells of PCOS rats; **J** Apoptosis of PCOS rat ovarian granulosa cells after MEG3 downregulation was detected by the TUNEL method (scale bar = 100 μm); green fluorescent FITC dye labelled apoptotic cells, blue fluorescent DAPI dye labelled nuclei, and Merge is a superimposed image showing both fluorescent signals; **K** fluorescence values of PCOS rat ovarian granulosa cells after MEG3 downregulation indicating apoptosis. D-K, N = 6. These data are expressed as the mean ± standard deviation, **P < 0.01 or ***P < 0.0001 compared to the normal group, ^##^*P* < 0.01 or ^###^*P* < 0.0001 compared to the sh-NC group and were statistically analysis using one-way ANOVA and Tukey’s post hoc test. 1: Normal group, 2: PCOS group, 3:sh-MEG3 group, 4:sh-NC group

**Fig. 6:**
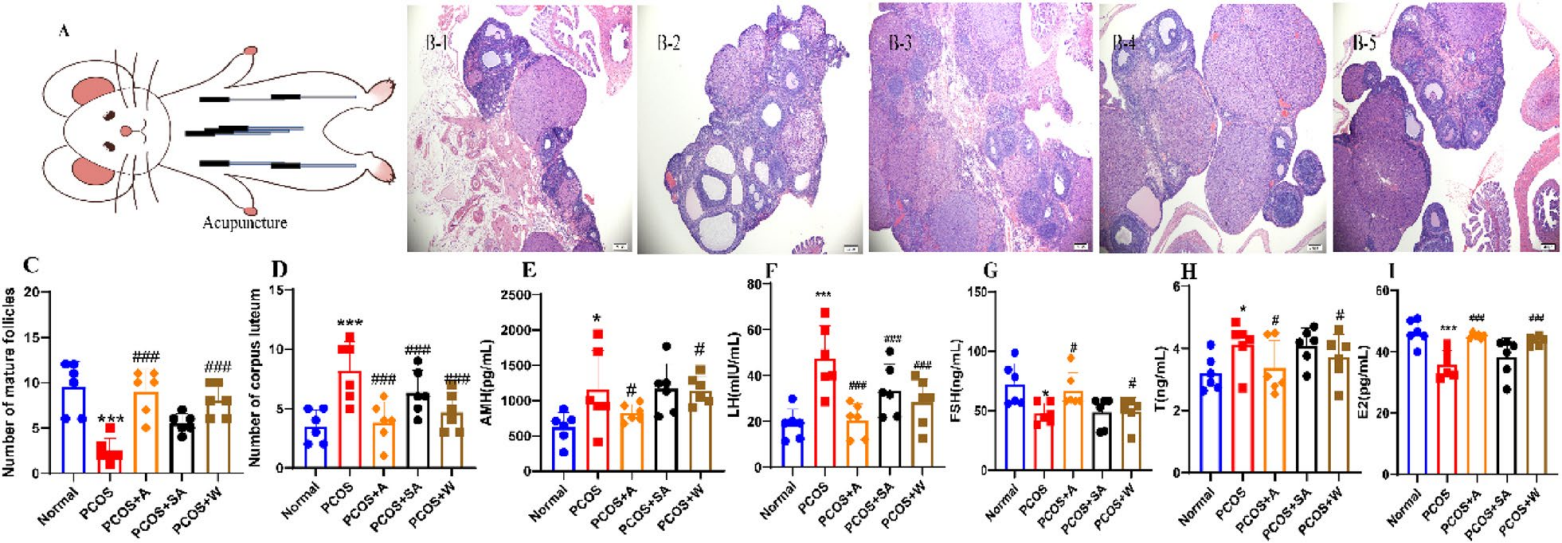
Acupuncture ameliorates abnormal sex hormone levels and ovarian histopathological changes in PCOS rats.** A** Experimental strategy for acupuncture intervention; **B** HE staining was used to observe pathological changes in the ovaries of PCOS rats after the acupuncture intervention (scale bar = 200 μm); **C** Number of mature follicles in the ovarian tissue of PCOS rats after the acupuncture intervention; **D** Number of corpus luteum structures in the ovarian tissue of PCOS rats after acupuncture intervention; **E** AMH secretion in the serum of PCOS rats after acupuncture intervention; **F** LH secretion in the serum of PCOS rats after acupuncture intervention; **G** FSH secretion in the serum of PCOS rats after acupuncture intervention; **H** T secretion in the serum of PCOS rats after acupuncture intervention; **I** E2 secretion in the serum of PCOS rats after acupuncture intervention. A-I, N = 6. These data are measured values, expressed as the mean ± standard deviation, * *P* < 0.05, *** *P* < 0. 0001 compared to the normal group; ^#^*P* < 0.05, ^###^*P* < 0.0001 compared to the PCOS group, statistically analysis using one-way ANOVA and Tukey’s post hoc test. E2 stands for oestradiol, AMH stands for anti-Mullerian hormone, T stands for testosterone, FSH stands for follicle-stimulating hormone and LH stands for luteinizing hormone. 1: Normal group, 2: PCOS group, 3: PCOS + A group, 4: PCOS + SA group, 5: PCOS + W group

To investigate the specific mechanism by which the MEG3/miR-21-3p axis is involved in the development of follicular abnormalities in PCOS, we used a rat model of PCOS induced by DHEA, along with silent MEG3 lentivirus intervention in PCOS rats (Figs. 2C, 3A, 4A, 5A, 6A). The expression of MEG3 (Fig. 2D) and miR-21-3p (Fig. 2E) was measured in the ovarian granulosa cells of rats with PCOS. The results showed that the expression of MEG3 and miR-21-3p was enhanced in the ovarian granulosa cells of the DHEA group compared with those of the normal group (*P* < 0.0001 for both). The expression of MEG3 and miR-21-3p was reduced in the sh-MEG3 group compared with the sh-NC group (both *P* < 0.0001).

### Silencing of MEG3 ameliorates abnormal sex hormone expression and ovarian histopathological changes in PCOS rats and promotes follicle cell development and maturation

In terms of histopathological changes in PCOS rats, ovarian granulosa cells in the normal group were observed to be intact and regularly arranged by HE staining. Fresh corpus luteum and follicles were observed and no pathological phenomena, such as cell necrosis, degeneration, or inflammatory infiltration were observed. Ovaries of rats in the PCOS and sh-NC groups showed diffuse cystic severe dilatation and typical polycystic changes. Most of the small follicles were dilated large follicles, with a few corpus luteum and mature follicles and incomplete granulosa cells. Sh-MEG3 rats showed diffuse cystic mild to moderate dilatation of the ovaries with a few corpus luteum and atretic follicles (Fig. 3B). The numbers of mature follicles and corpus luteum structures were increased in the sh-MEG3 group compared to the sh-NC group (*P* < 0.01) (Fig. 3C, D).

Abnormal follicular development and metabolic and endocrine disorders are the main pathological manifestations of PCOS, and hypothalamic gonadotropin-releasing hormone and ovarian oestrogen together regulate this process [23]. In this paper, the production of E2, AMH, T, FSH, and LH was measured by ELISA. The results showed that FSH and E2 production was decreased and T, AMH, and LH production was increased in the PCOS group compared to the normal control group (all *P* < 0.05). FSH and E2 production was increased and T, AMH, and LH production was decreased in the sh-MEG3 group compared to the sh-NC group (all *P* < 0.05) (Fig. 3E–I).

### Silencing MEG3 increases the number of granulosa cells

Granulosa cells (GCs) are a layer of epithelial cells that surround the oocyte and regulate follicular growth, development, and maturation in an autocrine and paracrine manner to maintain the corpus luteum. Granulosa cell dysfunction is frequently seen in PCOS and may play a role in its pathogenesis. After MEG3 silencing lentivirus intervention in PCOS rats, granulosa cells extracted from follicles were cultured in an incubator at 37 °C for 24 h. The growth of ovarian granulosa cells were observed after they adhered to the well. The results showed that the number of granulosa cells was lowest in the model group compared to the normal group (*P* < 0.0001) (Fig. 4A–C). There were more granulosa cells in the sh-MEG3 group than in the sh-NC and PCOS groups (*P* < 0.0001) (Fig. 4A–C).

### Silencing of MEG3 inhibits the apoptosis of ovarian granulosa cells in PCOS rats

Apoptosis of granulosa cells directly affects the development of oocytes. Under FSH stimulation, the gap junctions between granulosa cells and oocytes were in an open state, and molecular substances entered the oocytes in large quantities before the peak of LH secretion, which may be the result of apoptotic messages from granulosa cells to oocytes [23–25]. Our study assessed the expression of apoptotic proteins (including cleaved caspase-3, Bax and Bcl-2), mitochondrial membrane potential (JC-1), and TUNEL staining to detect early, mid and late stage apoptosis. After MEG3 silencing lentivirus intervention in PCOS rats, an increase in mitochondrial membrane potential (JC-1) could be detected in granulosa cells, so that the green fluorescence changed to red fluorescence (Fig. 5B, C). In turn, the shift of JC-1 from green to red fluorescence was detected as an early inhibition of apoptosis. In addition, the PCOS group showed increased increased caspase-3mRNA expression and cleaved caspase-3/caspase-3 ratio, decreased Bcl-2mRNA expression, increased Bax mRNA expression and decreased Bcl-2/Bax ratio to the normal group (both *P* < 0.01) (Fig. 5 D-I). Compared with the sh-NC and PCOS groups, the sh-MEG3 group showed decreased caspase-3 mRNA expression and decreased cleaved caspase-3/caspase-3 ratio, and increased Bcl-2mRNA expression, decreased Bax mRNA expression and increased Bcl-2/Bax ratio (all *P* < 0.01) (Fig. 5D–I). The results indicated that silencing MEG3 repaired the mitochondrial membrane potential and associated apoptotic proteins in the early stages of granulosa cell apoptosis. Furthermore, TUNEL assay of apoptotic genomic DNA breaks, fluorescence values of ovarian granulosa cells in the PCOS group were significantly higher than those in the normal control group (*P* < 0.01), indicating apoptosis, and the apoptotic cells were labelled. The fluorescence values of ovarian granulosa cells in the sh-MEG3 group were lower than those in the sh-NC group (*P* < 0.01), and a few apoptotic cells showed green fluorescence (Fig. 5J, K).

### Acupuncture improves polycystic ovarian morphology and sex hormone levels in PCOS rats

To further observe the histopathological alterations in PCOS rats after acupuncture intervention, ovarian granulosa cells in the normal group were observed to be intact and regularly arranged by HE staining (Fig. 6A). Fresh corpus luteum and follicles were observed, and no pathological phenomena, such as cell necrosis, degeneration, or inflammatory infiltration, were observed. Ovaries of rats in the PCOS and sh-NC groups showed diffuse cystic severe dilatation and typical polycystic changes. Most of the small follicles were dilated into large follicles, with a few corpus luteum and mature follicles and incomplete granulosa cells; the ovaries of the rats in the PCOS + A, PCOS + W groups showed diffuse cystic mild to moderate dilatation with a few corpus luteum and atretic follicles (Fig. 6B). The numbers of mature follicles and corpus luteum structures were increased in the PCOS + A group, the PCOS + W group compared with the PCOS groups (*P* < 0.0001) (Fig. 6C, D).

The abnormal secretion of FSH and LH from the pituitary gland, together with abnormal levels of E2, AMH, and T, promotes abnormal follicular cell development and endocrine disorders, which in turn affect polycystic changes in ovarian morphology. First, the production of E2, AMH, T, FSH, and LH in PCOS rats was measured by ELISA after acupuncture. The results showed that FSH and E2 production decreased and T, AMH, and LH production increased in the PCOS group compared with the normal group (all *P* < 0.05), while FSH and E2 production increased and T, AMH and LH production decreased in the PCOS + A and PCOS + W groups compared with the PCOS group (all *P* < 0.05) (Fig. 6E-I).

### Acupuncture intervention increases the number of granulosa cells

**Fig. 7:**
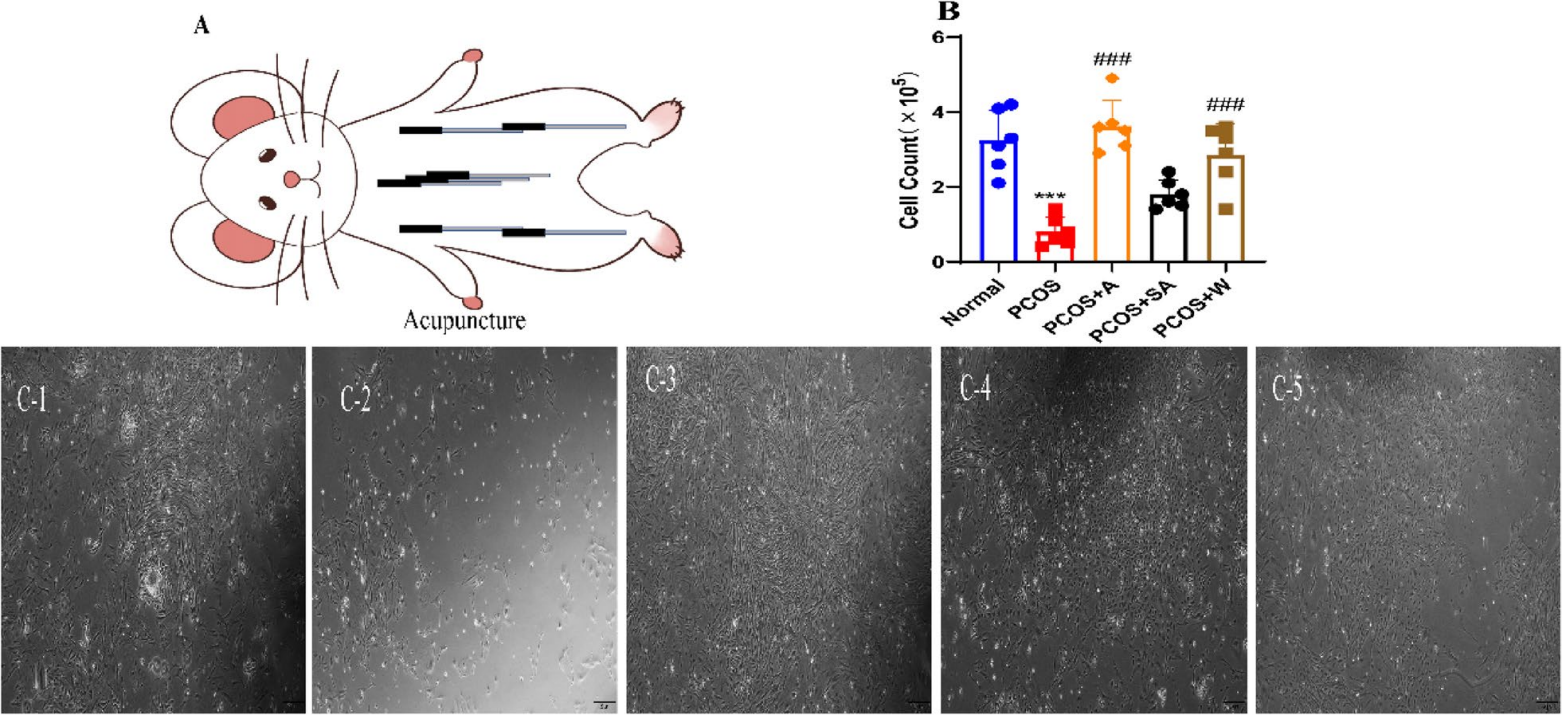
Acupuncture intervention in PCOS rats increases the number of granulocytes. **A** Experimental strategy for acupuncture intervention;** B** Number of granulosa cells in the ovaries of PCOS rats after acupuncture intervention; **C** Primary granulosa cells were extracted and cultured to observe cell number. The number of granulosa cells in the ovaries of PCOS rats were significantly increased after acupuncture intervention (scale bar = 100 μm). A-C, N = 6.These data are expressed as the mean ± standard deviation, ***P < 0.0001 compared to the normal group and ^####^*P* < 0.0001 compared to the sh-NC and PCOS groups. One-way factorial ANOVA and Tukey’s post hoc test were used for statistical analysis. 1: Normal group, 2: PCOS group, 3: PCOS + A group, 4: PCOS + SA group, 5: PCOS + W group

Ovarian granulosa cell apoptosis is essential in theprogression of PCOS. In this study, we investigated the increased number of granulosa cells in PCOS rats after acupuncture intervention by culturing primary granulosa cells, which were extracted from follicles and cultured in an incubator at 37 °C for 24 h (Fig. 7A). After adhering to the well, the ovarian granulosa cells were observed for growth. The results showed that the granulosa cells in the model group, sh-NC, were fewer in number than those in the normal group (*P* < 0.0001) (Fig. 7A, B). The number of granulosa cells were increased in the PCOS + A, PCOS + W groups compared to the PCOS groups (*P* < 0.0001) (Fig. 7B, C).

### Inhibition of apoptosis of ovarian granulosa cells in PCOS rats through LncMEG3-mediated regulation of miR-21-3p after acupuncture intervention

**Fig. 8:**
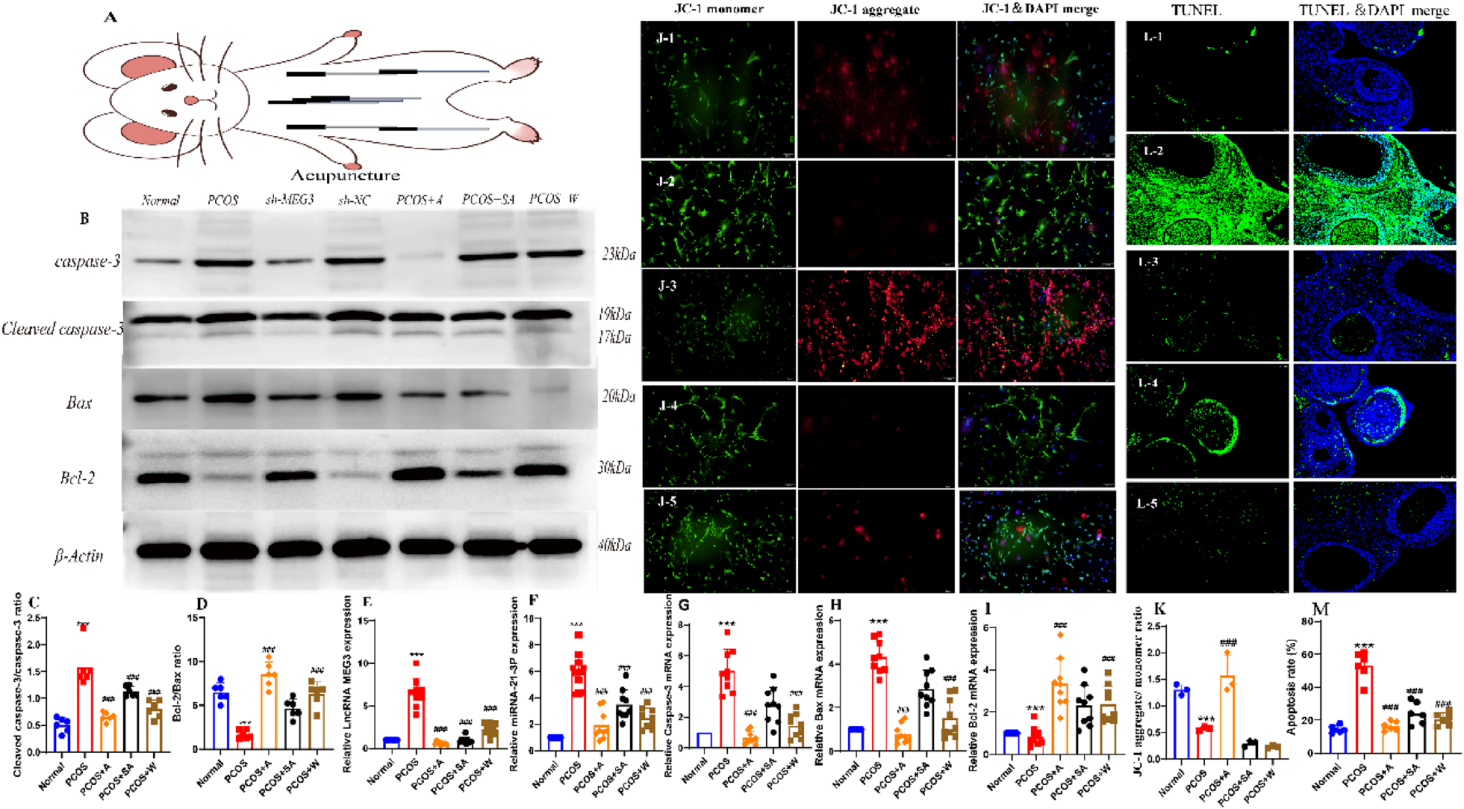
Inhibition of apoptosis of ovarian granulosa cells in PCOS rats through LncMEG3-mediated regulation of miR-21-3p after acupuncture intervention. **A** Experimental strategy for acupuncture intervention;** B** Cleaved caspase-3, caspase-3, Bax, and Bcl-2 protein bands in granulosa cells of PCOS rat ovaries after acupuncture intervention. **C** Protein blotting for cleaved caspase-3/caspase-3 protein expression in granulosa cells of PCOS rats after acupuncture intervention.** D** Western blotting for Bcl-2/Bax protein expression in granulosa cells of PCOS rats after needle prick intervention. **E** Expression of LncMEG3 in ovarian granulosa cells of PCOS rats detected by RT‒qPCR after acupuncture intervention. **F** Expression of miR-21-3p in ovarian granulosa cells of PCOS rats detected by RT‒qPCR after acupuncture intervention; **G** Detection of caspase-3 by RT‒qPCR in ovarian granulosa cells of PCOS rats; **H** Detection of Bax by RT‒qPCR in ovarian granulosa cells of PCOS rats; **I** Detection of Bcl-2 by RT‒qPCR in ovarian granulosa cells of PCOS rats;** B** The JC-1 method was used to detect the early stage of apoptosis in PCOS rat ovarian granulosa cells after MEG3 downregulation (scale bar = 100 μm): at high mitochondrial membrane potential, which can produce red fluorescence, at low mitochondrial membrane potential, which produces green fluorescence, and the blue fluorescent DAPI dye labels the nucleus, and Merge is a superimposed image showing the three fluorescent signals; **C** The ratio of JC-1 aggregate (red) to monomer (green) intensity in myotubes, Cell experiments were independently repeated three times; **L** Apoptosis of ovarian granulosa cells in PCOS rats after acupuncture intervention was detected by the TUNEL method (scale bar = 100 μm), with green fluorescent FITC dye labelling the apoptotic cells and blue fluorescent DAPI dye labelling the nuclei; the merged image is a superimposed image of both fluorescent signals; **M** Fluorescence values of ovarian granulosa cells in PCOS rats after acupuncture intervention reflecting apoptosis. B-M, N = 6. These data are expressed as the mean ± standard deviation, ***P < 0.0001 compared to the normal group and ^####^*P* < 0.0001 compared to the sh-NC group, using one-way ANOVA and Tukey's post hoc test for statistical analysis. 1: Normal group, 2: PCOS group, 3: PCOS + A group, 4: PCOS + SA group, 5: PCOS + W group

Acupuncture facilitates oocyte development by stimulating the secretion of FSH from the pituitary gland, closing the gap junctions between granulosa cells and oocytes, and inhibiting the entry of apoptotic molecular substances into oocytes in large quantities before the peak of LH secretion [35]. To further investigate the mechanism of action by which acupuncture inhibits the apoptosis of ovarian granulosa cells in rats with PCOS, we extracted primary ovarian granulosa cells from rats with PCOS after acupuncture intervention and detected the transcription of granulosa cell LncMEG3 and miR-21-3p, apoptotic proteins (including cleaved caspase-3, Bax and Bcl-2), mitochondrial membrane potential (JC-1), and TUNEL staining to detect early, mid and late-stage apoptosis. The transcription of LncMEG3, miR-21-3p, caspase-3, Bax and Bcl-2 was detected by RT‒qPCR, and the apoptosis of ovarian granulosa cells was detected by Western blot, and mitochondrial membrane potential (JC-1), and TUNEL assays. After acupuncture intervention in PCOS rats (Fig. 8A), the PCOS group showed increased caspase-3, LncMEG3 and miR-21-3p mRNA transcription, increased cleaved caspase-3/caspase-3 ratio, decreased Bcl-2 mRNA transcription, increased Bax mRNA transcription and decreased Bcl-2/Bax ratio compared to the normal group (all *P* < 0.0001) (Fig. 8B-I). Compared to the PCOS group, the PCOS + A, PCOS + W group showed decreased caspase-3 mRNA transcription, decreased cleaved caspase-3/caspase-3 ratio, increased Bcl-2 mRNA transcription, decreased Bax mRNA transcription and increased Bcl-2/Bax ratio (both *P* < 0.0001) (Fig. 8B–I). In addition, the PCOS + A, PCOS + W groups could detect an increase in mitochondrial membrane potential (JC-1) in granulosa cells, which changed green fluorescence to red fluorescence (both *P* < 0.0001) (Fig. 8J, K). Also, the shift of JC-1 from green to red fluorescence was detected as an early inhibition of apoptosis. The results indicated that pinning could repair granulosa cell mitochondrial membrane potential and associated apoptotic proteins in the early stages of granulosa cell apoptosis. In addition, TUNEL detection of apoptotic genomic DNA breaks showed significantly higher fluorescence values in ovarian granulosa cells in the PCOS group than in the normal control group (*P* < 0.0001), indicating apoptosis, and apoptotic cells were labelled. the PCOS + A, PCOS + W groups showed lower fluorescence values in ovarian granulosa cells than in the PCOS group (both *P* < 0.0001), and a few apoptotic cells showed green fluorescence (Fig. 8L, M), indicating that acupuncture regulates miR-21-3p via LncMEG3 to inhibit ovarian granulosa cell apoptosis in PCOS rats.

## Discussion

PCOS is one of the leading causes of infertility in women of reproductive age, with more than 50% of PCOS patients having severe follicular developmental abnormalities that can further affect the development of infertility [36]. In addition, women with PCOS have been reported to incur higher socioeconomic costs and experience more obstetric complications, including recurrent disease cycles, preeclampsia, gestational diabetes, and preterm birth [37], making prevention and treatment of follicular developmental abnormalities key to the treatment of PCOS. The disease is mostly treated clinically with pharmacological ovulation promotion or Western surgery, but there are many adverse effects, the disease reoccurs easily after discontinuation of medication or surgery, and the long-term outcome is poor. Acupuncture can also be used to treat various clinical symptoms of polycystic ovary syndrome and its effectiveness and safety have been proven in clinical practice, with some studies finding that acupuncture is mainly effective in improving follicular development and ovarian function in patients with polycystic ovary syndrome [35, 38, 39]. In this study, we selected acupuncture points based on the integratin of clinical experience based on the understanding of PCOS according to TCM theory with meridian selection analysis [31]; we explored in depth the role of LncMEG3 and miR-21-3p in the apoptosis of ovarian granulosa cells in PCOS and the potential mechanism of acupuncture in treating abnormal follicular development in PCOS. We found that acupuncture stimulated the proliferation and inhibited the apoptosis of PCOS granulosa cells through LncMEG3-mediated downregulation of miR-21-3p to improve follicular development in PCOS rats.

The preliminary results of our study showed that LncMEG3 and miR-21-3p were highly expressed in PCOS samples. In line with our findings, a recent study highlighted that LncMEG3 expression tends to be upregulated in the context of PCOS [19]. Similarly, LncMEG3 was upregulated in PCOS tissues and cells [19, 40]. Similar to our study, previous studies have shown that miR-21 is highly expressed in the serum of ovarian cancer patients and leads to enhanced proliferation, migration, and invasion of neoplastic cells, which may be of high clinical value for the diagnosis of ovarian cancer. However, the relationship between the expression of LncMEG3 and miR-21-3p in PCOS has rarely been studied. Furthermore, miR-21 expression levels were significantly higher in the serum of obese PCOS patients than in controls [41]. Moreover, it has been shown that MEG3 can act as competing endogenous RNA (ceRNA) to adsorb miR-21, while the targeting relationship between MEG3 and miR-21-3p in PCOS needs further validation.

Next, we found that silencing LncMEG3 led to the downregulation of miR-21-3p, thereby promoting FSH and E2 production; inhibiting T, AMH and LH production; and suppressing apoptosis in PCOS rat ovarian granulosa cells. We assessed apoptotic protein family (including cleaved caspase-3, Bax and Bcl-2) expression, mitochondrial membrane potential, and TUNEL staining to assess early, middle and late-stage apoptosis [42, 43], as early and late apoptosis of granulosa cells is an important pathological factor in abnormal follicular development related to PCOS. The literature has found that silencing LncMEG3 promoted fine cell proliferation, PCNA expression and cyclin D1 expression [44], which is consistent with our findings that ectopic expression of MEG3 stimulates the development of PCOS and that inhibits ovarian granulosa cell proliferation and prevents apoptosis [19]. Furthermore, a previous article indicated that overexpression of MEG3 inhibited the proliferation, invasion and migration of pituitary tumour cells and accelerated apoptosis [45]. On the other hand, miR-21 is closely associated with metabolic disorders, insulin sensitivity and ovarian pathological processes [15]. Moreover, miR-21 is significantly upregulated in the serum of PCOS patients vs. controls, and it is hypothesized that miR-21 expression has potential applications for the diagnosis and treatment of PCOS [46]. In addition, LncMEG3 exerts tumour-suppressive effects mainly by regulating complex and diverse processes such as cell proliferation, differentiation, apoptosis and signal transduction, providing new ideas and approaches for tumour diagnosis and treatment. miR-21 is also an important regulator of bone regeneration and various bone diseases [47]. All of the above evidence confirms the positive role of miR-21 expression and apoptosis, which are dysregulated but returned to normal levels by LncMEG3, in disease development.

In PCOS, acupuncture therapy has been shown to have significant therapeutic effects [48–50]. However, the exact mechanism of acupuncture for the treatment of PCOS is not well understood [51]. LncMEG3 has been reported to be upregulated in granulosa cells in the levonorgestrel 18 methyl nortriptyline silicone capsule-induced PCOS rat model, and inhibition of LncMEG3 expression by acupuncture can alleviate PCOS, as evidenced by reduced T, LH and FSH secretion, as well as impaired granulosa cell proliferation and autophagy [19]. In a rat model of PCOS with levulinic 18 methyl kynurenine in silico capsules, abnormal granulosa cell LncMEG3 expression was sufficient to cause cystic ovarian changes in rats, but the abnormal changes in sex hormone levels and AMH induced by levulinic 18 methyl kynurenine in silico capsules in a rat model of PCOS did not fully account for the clinical manifestations of clinical PCOS, and it has not yet been explored what genes downstream of LncMEG3 targeting regulation are involved. The abnormal expression of LncMEG3 in granulosa cells is not sufficient to clarify its importance in other reproductive phenotypes of PCOS disease [52]. In the present study, we focused on the phenotypic changes in reproduction in rats with DHEA-induced PCOS. In addition, low expression of LncMEG3 in ovarian cancer promoted tumour cell proliferation and inhibited tumour cell apoptosis. From another perspective, acupuncture can regulate FSH and LH, promote granulosa cell proliferation and inhibit apoptosis, thereby improving follicular development abnormalities in PCOS [35, 53]. The cell goes from an early stage of apoptosis involving mainly intracellular changes in some proteins to an intermediate and late stage of apoptosis accompanied by DNA breakage and the formation of apoptotic vesicles. The main involvement of acupuncture in inhibiting apoptosis in the apoptosis cycle is also the subject of further investigation in this study. In conclusion, acupuncture silences LncMEG3 to inhibit apoptosis and attenuate follicular development abnormalities in PCOS.

**Fig. 9:**
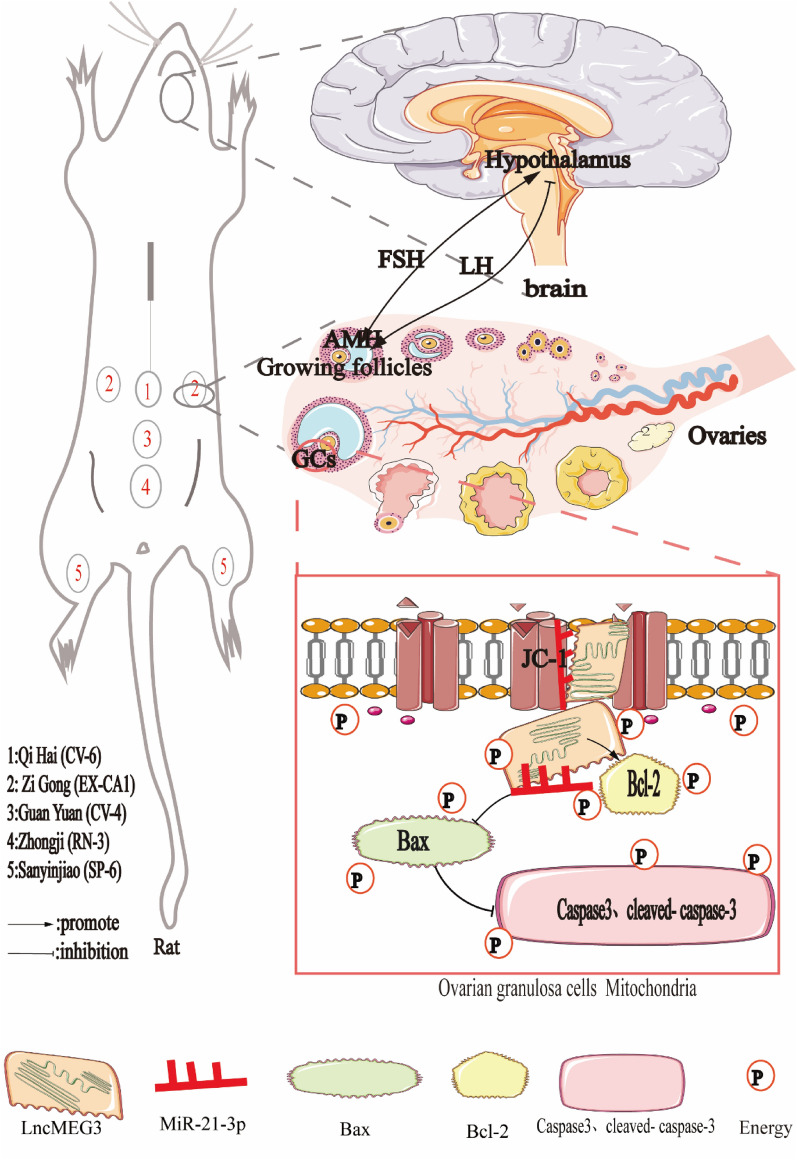
Acupuncture regulates the apoptosis of ovarian granulosa cells in the context of abnormal follicular development in PCOS through LncMEG3-mediated inhibition of miR-21-3p

Our results showed that acupuncture treatment significantly inhibited DHEA-induced LncMEG3 expression and that overexpression of LncMEG3 impaired the protective effect of acupuncture in rats with a PCOS model. In addition, LncMEG3 was involved in the inhibitory effect of acupuncture on ovarian granulosa cell apoptosis (whose apoptosis involves protein and DNA repair in the early and late stages of apoptosis) in rats with PCOS, thus ameliorating abnormal follicle development. In rats with PCOS, acupuncture inhibited miR-21-3p expression by targeting LncMEG3, while LncMEG3 silencing targeted the activation of miR-21-3p expression in ovarian granulosa cells, promoting FSH and E2 production and inhibiting T, AMH and LH production, thereby inhibiting early and late apoptosis in ovarian granulosa cells and improving abnormal follicle development (Fig. 9). Thus, our findings further elucidate a new mechanism by which acupuncture treats abnormal follicle development in PCOS.

Although our study reveals the role and mechanism of the LncMEG3/miR-21-3p axis in the development of PCOS, there are some limitations. We only investigated the effect of acupuncture on the abnormal expression of the LncMEG3/miR-21-3p axis in ovarian granulosa cells of PCOS rats, but it is unclear whether acupuncture-mediated regulation of LncMEG3/miR-21-3p axis levels affect metabolic phenotypes such as obesity and insulin resistance in PCOS. While this study detected mitochondrial membrane potential (JC-1) and early apoptosis, whether acupuncture affects the mitochondrial apoptotic pathway on the LncMEG3/miR-21-3p axis in ovarian granulosa cells of PCOS rats is not known. Therefore, we plan to conduct basic research to determine whether acupuncture regulates the LncMEG3/miR-21-3p axis and whether it affects the mitochondrial apoptotic pathway to treat metabolic phenotypes such as obesity and insulin resistance in PCOS, providing a new experimental basis for the treatment of PCOS with acupuncture. The clinical study was also designed to collect blood samples and follicles from patients with PCOS and after acupuncture intervention and to examine the LncMEG3 levels in patients' blood samples and follicles. The combination of basic and clinical studies will play an important role in guiding the clinical application of acupuncture in the treatment of PCOS.

## Conclusion

Acupuncture promotes PCOS granulosa cell proliferation and inhibits early and late apoptosis by downregulating LncMEG3, possibly by targeting and regulating miR-21 expression. It is suggested that the LncMEG3/miR-21 axis plays an important role in the pathogenesis of PCOS and that acupuncture treats abnormal follicular development in PCOS by regulating the LncMEG3/miR-21 axis. This study provides new possible molecular targets for acupuncture in the treatment of PCOS and provides a new theoretical basis for subsequent drug development. However, this study was partly limited to basic research, and a clinical validation study is needed in the future.

## Data Availability

The datasets used or analyzed during the current study are available from the corresponding author on reasonable request.
